# Around the Hospitals

**Published:** 1903-09-26

**Authors:** 


### AROUND THE HOSPITALS.

### THE ITALIAN HOSPITAL.

All the beds in this hospital are now open, and will be so throughout the current year. The number of in-patients treated last year was 600; out-patients, 10,111. Nearly 5,000 of the total number were British. Of the in-patients 443 were Italian, 121 British, 13 Swiss, and 9 German, while other nationalities represented were French, Austrian, Spanish, Russian, Dutch, Bavarian, Algerian, Indian, and Armenian. There were 25 deaths. The average number of days' residence was 20·83. Among the out-patients were 4,809 British, 4,053 Italians, 453 Germans, 234 Poles, 206 Russians, and 104 Frenchmen, while there were a few Austro-Hungarians, Spaniards, Armenians, Greeks, and Indians. Annual subscriptions amounted to £344 (a slight increase on the previous year); the Italian Government gave its grant of £100, and the Swiss Government £7 18s. 4d. Donations were nearly £500 more than in 1901, and the total ordinary income was £3,418, an increase of £1,819. There was, however, only a small legacy of £152, as compared with last year's special collection for endowing a bed in memory of the late King Humbert. The total expenditure was £2,471, of which £120 was extraordinary, being an appeal against assessment. No analysis of the cost per patient is given in the report, nor is there a medical and surgical report showing the nature of the cases treated as in-patients.

### QUEEN CHARLOTTE'S HOSPITAL: THE YEAR'S WORK.

The number of in-patients during 1902 was 1,271, and the daily average number of beds occupied 46. The figures are slightly below those of 1901. Five deaths occurred, two from heart disease, one from eclampsia, two from septicæmia. Of the last two, one was admitted with the disease, while the other developed it as a result of the disease from which she was suffering on admission. In the out-patient department there were 1,204 patients, an increase of 178 on the previous year; one death occurred from consumption, from which the patient had long suffered. An important alteration has been made in the attendance given to the out-patients, who are now daily visited, both mother and child being attended to for at least nine days. Formerly only occasional attendance was given to the mother. The extra assistance is much appreciated.

The ordinary income was £3,771. Annual subscriptions showed an increase of £110, but donations fell by £339. The Hospital Sunday Fund gave £479 3s. 4d., and the Hospital Saturday Fund £131 6s. Legacies were £1,808 15s. 1d. The total ordinary expenditure was £5,183, an increase of £93. This increase is apparent in almost every section, except in rates and taxes, insurance, and appeals. Additional support is needed for the convalescent home; 123 patients were received, the income was £330, and the expenditure £418. A special donation of £50 from King Edward's Fund is included in the income, and the deficit of £88 was met by drawing on the Samaritan Fund.

### THE ROYAL FREE HOSPITAL.

#### The Year's Work.

The Royal Free Hospital received during the year 43,000 patients—the largest number yet dealt with, and 3,500 more than during 1901. Two thousand six hundred and thirty-five of the sick and suffering people were treated in the in-patient wards, for an average of 20 days each, entirely without charge. The hospital also relieved 17,392 out-patients; while 23,000 casualty cases—63 for every day of the year including Sundays—came in from accidents. Most of these patients came from the immediate neighbourhood. It has been stated that 23 per cent. of the patients in the metropolitan hospitals came from the country, but that was not the experience at the Royal Free. In its case only 9 per cent. came from the provinces, thus showing the need for the hospital in the district in which it is situated. In spite of the large increase in the work done, the ordinary expenditure, amounting to £13,489, was only £400 more than in 1901. The income for 1902 was the largest for many years, probably the largest in the history of the institution. No less than £12,000 was received in legacies, making with the ordinary income a total of £19,000 for the year. Of this £500 was put to reserve.

